# Point-of-care ultrasound during trauma and critical care transport: a practical, safety-gated review

**DOI:** 10.3389/fpubh.2026.1816318

**Published:** 2026-05-21

**Authors:** Lihui Chen, Jiajia Li, Xiuxiu Chen, Jie Huang, Jialu Liang, Chenying Qu, Yaling Jin, Yangtian Ye, Mengqi Cai, Weiting Chen

**Affiliations:** 1 Department of Emergency Medicine and Intensive Care Medicine, The First People's Hospital of Linhai, Taizhou, Zhejiang, China; 2 Department of Clinical Medicine, Hangzhou Medical College, Hangzhou, Zhejiang, China

**Keywords:** aeromedical transport, cardiac arrest, critical care air transport team, critical care retrieval, EFAST, helicopter emergency medical services, interhospital transport, lung ultrasound

## Abstract

**Background:**

Air medical and critical care transport teams often treat severely injured or critically ill patients when imaging, laboratory testing, and specialist support are limited. Point-of-care ultrasound (POCUS) may support early recognition of reversible conditions, but its value depends on strict indication selection, operator competence, and interpretation rules that prevent false reassurance.

**Methods:**

This structured narrative review summarizes evidence and practical considerations for transport POCUS in trauma and critical care retrieval. The review is organized around a safety-gated framework and groups evidence by clinical use: extended FAST (eFAST) and lung ultrasound, shock assessment, cardiac arrest POCUS, ultrasound-guided procedures, workflow integration, governance, tele-mentoring, and research priorities.

**Main findings:**

Transport POCUS is most useful when it confirms a limited set of urgent, actionable findings. eFAST and thoracic ultrasound generally show high specificity but only moderate sensitivity; therefore, a clear positive finding may trigger action, whereas a negative or technically limited scan should not justify down-triage. Representative studies suggest that prehospital POCUS can influence care processes: one randomized multicenter trial reported that prehospital FAST reduced the median time to admission by 13 min and the median time to operative treatment by 15 min, while a prospective HEMS cohort found that POCUS was used in 34.5% of patients and had therapeutic consequences in 40.7%. However, evidence for mortality, complication reduction, and long-term patient-centered outcomes remains limited.

**Conclusion:**

Transport POCUS should function as a focused decision-support tool, not as a substitute for definitive imaging. Safe implementation requires clear scope, time caps, “indeterminate = not reassuring” rules, structured documentation, competency-based training, discrepancy review, and quality assurance. Future studies should move beyond feasibility and time-to-care endpoints to evaluate safety, false-negative harms, complications, and patient-centered outcomes.

## 1 Background and rationale

Transport teams care for patients whose condition can change within minutes, but diagnostic imaging options are limited during flight, ambulance transfer, and urgent interfacility retrieval. Some life-threatening problems are reversible if teams recognize them early. Tension pneumothorax, major internal bleeding, obstructive shock, and cardiac tamponade are typical examples. In this context, bedside tools that provide rapid and usable information may help teams act sooner, communicate with receiving hospitals, and choose a destination that matches the patient’s physiology and likely intervention needs (1–4).

FAST became part of trauma care through international consensus, and eFAST later added thoracic views for pneumothorax and hemothorax (5, 6). This matters during transport because CT is unavailable and radiography is usually impractical. Evidence summaries suggest that prehospital ultrasound can change care decisions, including treatment steps, destination choice, and activation of hospital resources (7–9). More recent pathway studies also suggest that prehospital FAST can shorten selected time-to-care metrics when it is embedded in a mature trauma system (10–16).

The transport environment also increases the risk of misinterpretation. Motion, vibration, limited patient access, dim or variable lighting, restricted workspace, and competing airway, ventilation, and hemorrhage-control tasks can reduce image quality and increase cognitive load (10, 11, 17). A meta-analysis reported that eFAST has high specificity but only moderate sensitivity in trauma patients (18). Transport POCUS should therefore be framed as a decision-support tool for actionable positive findings rather than as a replacement for definitive imaging. A focused scan is useful only when the image quality and clinical context are strong enough to trigger a predefined action; otherwise, the finding should be labeled indeterminate and treated as not reassuring (17, 18).

## 2 Methods and scope

This article is a structured narrative review, not a systematic review or meta-analysis. Because transport POCUS evidence includes diagnostic accuracy studies, feasibility studies, prehospital cohort studies, implementation reports, tele-mentoring studies, and guidance documents, a narrative synthesis was considered more appropriate than pooled quantitative analysis.

The literature was organized using a structured search-and-mapping approach. For this review, transport POCUS refers to focused ultrasound performed during prehospital care, HEMS missions, interfacility retrieval, critical care transport, military or CCATT-style transport, or immediately before or during transfer when the result can influence transport decisions. Relevant studies and guidance documents were identified through targeted searches of PubMed/MEDLINE, Embase, Web of Science, Scopus, Google Scholar, and reference lists from key reviews. Search terms included combinations of point-of-care ultrasound, POCUS, prehospital ultrasound, transport, aeromedical transport, helicopter emergency medical services, HEMS, interfacility transport, critical care retrieval, CCATT, FAST, eFAST, lung ultrasound, BLUE protocol, RUSH protocol, shock, cardiac arrest, ultrasound-guided procedure, vascular access, tele-mentoring, governance, training, and quality assurance. The search was updated to March 2026.

Eligible sources included English-language studies and guidance documents addressing POCUS in prehospital, HEMS, interfacility retrieval, military or CCATT-style, and transport-relevant emergency and critical care contexts. Hospital-only studies were included only when they informed transport-relevant interpretation, training, protocol design, or safety limitations. Recent evidence from the past 5 years was prioritized where available, while older landmark FAST, eFAST, BLUE, RUSH, transport, and lung ultrasound references were retained when they established definitions or widely used protocol concepts (5, 6, 19–21).

Data were extracted narratively for clinical setting, ultrasound application, study design, sample size, operator group, scan duration or feasibility, diagnostic performance, management change, time-to-care effects, patient-centered outcomes, and reported limitations. A formal risk-of-bias assessment was not performed. Instead, evidence strength was judged according to study design, sample size, transport specificity, outcome type, and whether the findings could plausibly be separated from broader system-level changes. This approach was used to avoid overinterpreting feasibility or pathway studies as proof of mortality benefit.

## 3 Operational constraints unique to transport POCUS

Transport POCUS faces recurring limits that can reduce image quality and change how the result should be used. Together, these constraints increase the likelihood of indeterminate scans and support time caps, stop rules, and a strict rule-in approach.

Image noise from motion and vibration can mimic or hide real findings. Teams often need short, stable clips and cine loops rather than prolonged scanning. When lung sliding is difficult to judge on B-mode, M-mode may help; true sliding tends to show a seashore pattern, whereas absent sliding tends to show a barcode pattern (22).

Short time windows are typical. Scanning usually occurs before loading, soon after departure, during a stable transport phase, or just before handover. A scan should not compete with airway control, ventilation, external hemorrhage control, blood product administration, vasopressor support, or other immediate resuscitation tasks. Transport guidance supports clear role assignment so that one clinician can scan only when the rest of the team can continue essential care (1–3).

Patient access is often limited by harnesses, immobilization devices, cabin layout, surgical dressings, body habitus, and pelvic binders. Pelvic views and posterior lung views are often the most difficult. A pelvic binder may obscure the suprapubic FAST window; teams can scan just above the binder edge but should not loosen a binder only to improve an ultrasound window.

High cognitive load is a major safety issue. Scan interpretation can distract from resuscitation, and fixation on an equivocal finding can create harm. A short checklist, predetermined action triggers, and documentation of confidence and limitations can reduce this risk (17, 23, 24). If images are not clearly interpretable, the scan should be documented as indeterminate and should not delay transport or definitive imaging.

## 4 Evidence base and critical appraisal

The evidence base for transport POCUS is clinically promising but methodologically uneven. Many studies evaluate feasibility, scan duration, diagnostic agreement, or management change rather than mortality, complications, neurological outcomes, or long-term functional outcomes. Transport outcomes are also highly system dependent. Staffing model, dispatch criteria, receiving-hospital capability, trauma activation rules, blood availability, and transport distance can all modify the effect of ultrasound. Therefore, available studies should be interpreted as evidence that POCUS can support pathway-level decisions, not as proof that ultrasound alone improves survival (Table 1).

**Table 1:** Transport POCUS applications in trauma, shock, respiratory failure, cardiac arrest, and procedures: actionable rule-in findings and escalation rules.

Clinical question	Minimum views or action	Actionable positive (“rule-in”)	What the team can do during transport	If negative or unclear	Common pitfalls/notes
Is there intraperitoneal free fluid suggesting major bleeding?	FAST RUQ, LUQ, pelvic views (≤60–90 s total)	Free fluid in any window, especially with shock	Pre-alert trauma/OR/IR, prepare blood, confirm destination	Do not down-triage. Continue bleeding-control pathway. Repeat only if physiology changes. Confirm with definitive imaging.	Early bleeding and retroperitoneal bleeding may be missed. Pelvic binder and positioning can limit pelvic view. eFAST confirms rather than excludes injury (18).
Is there pneumothorax that may need decompression?	Anterior thorax bilaterally (≤30–60 s)	Absent sliding with supportive signs in context; lung point when seen	If unstable and suspicion is high, follow decompression protocol and reassess	Treat as uncertain if not clearly positive. Manage clinically and confirm on arrival. Repeat if conditions change.	Subcutaneous emphysema and dressings block windows. Low tidal volume, apnea, mainstem intubation, or pleural adhesions can reduce sliding (19, 25, 26).
Is there hemothorax or pleural fluid worsening ventilation?	Basal or posterolateral view if accessible	Pleural collection consistent with effusion/hemothorax	Inform receiving team. Plan timing of chest tube, often at destination unless instability requires action.	Use clinical trajectory and destination imaging when views are limited.	Posterior views are often difficult during transport (26).
Is there pericardial effusion or tamponade concern?	Subxiphoid or other feasible cardiac view (≤20–30 s)	Effusion with compatible shock or arrest physiology	Inform destination and activate thoracotomy/tamponade pathway where appropriate	Do not exclude if negative or unclear. Continue standard trauma/shock/arrest algorithm.	Avoid long scans during CPR. Keep pause time strict (17, 28).
Is shock likely obstructive, cardiogenic, hypovolemic, or distributive?	Short RUSH-adapted view set: focused cardiac, lung, abdomen, IVC/venous assessment only if feasible	Severe LV dysfunction, marked RV dilation in context, pericardial effusion, free fluid, gross venous congestion or depletion	Adjust fluid/vasopressor strategy, prepare blood, activate PE/tamponade/cardiogenic shock pathway, guide destination choice	Do not exclude shock cause. Continue resuscitation and definitive assessment.	A complete RUSH exam is often unrealistic in transport; use question-first scanning (21).
Is respiratory failure explained by lung ultrasound findings?	Limited BLUE/LUS-adapted bilateral accessible lung zones	Clear pneumothorax pattern, diffuse B-lines, focal consolidation, large pleural effusion	Adjust ventilation strategy, assess fluid tolerance, communicate likely cause to destination	Treat as uncertain if images are incomplete or patterns do not fit clinical findings.	B-lines and consolidation are not disease-specific; integrate with physiology and history (20).
Can POCUS support cardiac arrest management?	Single focused cardiac view during planned pulse/rhythm check; lung view only if feasible	Tamponade, severe RV dilation in context, cardiac activity or standstill, pneumothorax signs	Support reversible-cause algorithm and receiving-team activation	Do not prolong CPR interruptions. Do not use POCUS alone to terminate resuscitation.	Probe and screen must be ready before the pause; POCUS must not prolong compressions (28).
Is ultrasound-guided vascular access feasible?	Target vessel short-axis or long-axis view before loading or during stable phase	Visible target vessel and safe needle path	Facilitate difficult peripheral/long peripheral/arterial access when clinically needed	Use alternative access routes if scanning delays urgent access.	Do not delay intraosseous access when immediate access is required. Sterility and motion matter (29–31).
Should the team repeat scans?	Trigger-based repeat of key views	New clear positive finding or clear change after intervention	Check response after decompression/fluids/ventilation changes and improve handover accuracy	If still non-diagnostic, stop and document limits. Escalate to definitive imaging.	Repeat only when the result can change near-term action (17).

A practical synthesis of the available literature supports four points. First, eFAST and thoracic ultrasound are most defensible as rule-in tools because specificity is generally high, while sensitivity is less reliable (18, 19, 25, 26). Second, POCUS can change management in selected prehospital and HEMS cohorts. In a prospective Dutch HEMS cohort, POCUS was used in 34.5% of patients and had therapeutic consequences in 40.7%; the effect was 26.0% among trauma patients, 65.4% among non-trauma patients, and 75.7% among patients undergoing CPR (16). Third, selected pathway studies report time-to-care advantages. Lucas et al. (13) reported that adding prehospital FAST reduced the median time to admission by 13 min and to operative treatment by 15 min, while Gamberini et al. reported shorter door-to-CT or door-to-OR time in patients with positive prehospital FAST (46 vs. 69 min) (12). Fourth, the link between these process outcomes and patient-centered outcomes remains uncertain.

Recent evidence also highlights why negative scans should be interpreted cautiously. In a 2025 diagnostic study of newly trained emergency physicians, eFAST showed 100% specificity but lower sensitivity for thoracic findings: 35.71% for hemothorax and 59.38% for pneumothorax, with false-negative cases sometimes requiring chest tubes after subsequent clinical deterioration or mechanical ventilation (27). Although this study was ED-based rather than transport-based, it reinforces a transport-relevant safety principle: a negative or technically limited scan should not be treated as reassurance when the clinical trajectory remains concerning.

Table 2 summarizes representative quantitative evidence. Overall, these data support transport POCUS as a structured decision aid while underscoring the need for future studies to report indeterminate rates, false-negative harms, scan-related delays, discrepancies with CT or operative findings, procedure-related complications, and patient-centered outcomes. Accordingly, the clinical sections that follow begin with trauma-focused applications and then extend to shock, respiratory failure, cardiac arrest, procedural support, and implementation.

**Table 2:** Representative quantitative evidence relevant to transport and prehospital POCUS.

Study	Setting/design	Sample/protocol	Key quantitative findings	Clinical implication	Main limitation
Lucas et al. (2022) (13)	Prospective randomized multicenter prehospital trauma trial	296 assessed trauma patients; 242 analyzed as treated (100 clinical exam only, 142 clinical exam + prehospital FAST)	CEX-p-FAST sensitivity 94.7% and specificity 97.6% for free fluid; median time to admission reduced by 13 min; median time to operative treatment reduced by 15 min; crossover 30.8%.	Supports pathway-level time benefit when FAST is integrated into prehospital trauma workflow.	Not powered to prove mortality benefit; crossover and system effects complicate causal interpretation.
Gamberini et al. (2022) (12)	Retrospective observational abdominal trauma study	199 patients with moderate/severe liver or spleen injury; 44 underwent prehospital FAST; 17 prehospital FAST-positive (27 ED FAST-positive within the prehospital FAST subgroup)	Prehospital FAST sensitivity 62.9% and specificity 100%; positive prehospital FAST associated with shorter door-to-CT or door-to-OR time (46 vs. 69 min, *p* < 0.001).	Suggests positive prehospital FAST can accelerate definitive care in established trauma systems.	Selected population and system-specific pathway; outcome benefit not directly proven.
Vianen et al. (2023) (16)	Prospective Dutch HEMS cohort	209 patients analyzed after POCUS; trauma, non-trauma, and CPR cases	POCUS used in 34.5% of HEMS patients; median scan time 3 min; median on-scene prolongation 0 min; therapeutic consequence in 40.7% overall, 26.0% trauma, 65.4% non-trauma, and 75.7% CPR cases.	Shows POCUS may affect treatment, destination, CPR decisions, and evaluation of treatment effect.	Single-system physician HEMS cohort; patient outcome effect unclear.
Smith et al. 2024 (39)	Retrospective HEMS case series after eFAST implementation	655 eligible blunt/penetrating trauma cases; 258 received prehospital ultrasound	eFAST changed clinical care in 7/258 scans (2.7%; 95% CI 1.1–5.5%).	Shows low-frequency but potentially high-impact management changes in HEMS trauma care.	Retrospective design; management-change endpoint, not patient-centered outcome.
Engelsen et al. 2024 (37)	In-flight telementored eFAST feasibility study	Ambulance helicopter telementoring protocol	Remote expert rated image quality mean 4.9/5; mean exam duration 05:54 min; LTE coverage depended on flight path and altitude and could fail above 2000 ft.	Tele-mentoring is feasible but requires dropout plans and fallback workflows.	Feasibility study; communication reliability limits generalizability.
Buyurgan et al. 2025 (27)	Prospective ED diagnostic study by newly trained emergency physicians	158 adult multiple-trauma patients; E-FAST vs. CT	Hemoperitoneum sensitivity 81.25%, specificity 100%; hemothorax sensitivity 35.71%, specificity 100%; pneumothorax sensitivity 59.38%, specificity 100%; false-negative thoracic cases sometimes required chest tube after clinical deterioration or ventilation need.	Reinforces high-specificity, limited-sensitivity pattern and the need for “negative does not reassure” rules.	ED rather than transport setting; single assessment; operator training and protocol details affect accuracy.

## 5 Trauma-focused transport POCUS: eFAST, pneumothorax, hemothorax, and free fluid

Trauma remains the most established use case for transport POCUS. The strongest transport role is not exclusion of injury, but confirmation of actionable problems when images are technically adequate and the finding fits the clinical picture.

For intraperitoneal free fluid, a clear positive FAST in a patient with shock can support early activation of a hemorrhage pathway, destination confirmation, blood product preparation, OR interventional radiology pre-alert, and direct communication with the receiving trauma team (7–14, 18). However, early bleeding, retroperitoneal bleeding, bowel injury, and technically limited pelvic views may be missed. A negative scan should therefore not stop hemorrhage control, trauma activation, CT on arrival, or surgical evaluation when the physiology remains concerning (17, 18).

For pneumothorax, lung ultrasound has high specificity in meta-analyses and guideline summaries (19, 25, 26). Clear positive findings may support decompression in an unstable or ventilated trauma patient when the clinical context is compatible. The lung point, when present, strongly supports pneumothorax (19). However, absent lung sliding alone is not specific; it may occur with apnea, mainstem intubation, low tidal volume, pleural adhesions, and other causes. Subcutaneous emphysema, chest dressings, and limited access can also prevent adequate visualization. If the pleural line is not assessable, the scan should be documented as indeterminate, and the team should manage according to clinical concern and destination imaging plans (17, 19, 25, 26).

For hemothorax or pleural fluid, posterior or basal views may be difficult during transport. When a pleural collection is clearly seen and ventilation or shock is deteriorating, the result can improve pre-arrival communication and procedural planning. In many systems, definitive tube thoracostomy may still be performed at the destination unless instability requires immediate action.

## 6 Beyond eFAST: shock, respiratory failure, and cardiac arrest POCUS

Transport POCUS should extend beyond eFAST because transport teams also encounter undifferentiated shock, respiratory failure, and cardiac arrest. eFAST remains the core trauma module, whereas RUSH-type shock assessment, BLUE-type lung ultrasound, focused cardiac ultrasound, and lung ultrasound can inform time-critical decisions when simplified for the transport environment (20, 21, 28–31).

RUSH-type assessment is useful when shock physiology is unclear, and the result can change immediate action. In RUSH-type assessment, ultrasound is organized around the pump, tank, and pipes (21). During transport, a complete RUSH exam may be unrealistic. A safer adaptation is a short, question-first scan: Is there pericardial effusion with compatible physiology? Is severe left ventricular dysfunction likely? Is marked right ventricular dilation present in a context suggesting massive pulmonary embolism? Is there free fluid suggesting hemorrhage? Are there gross signs of volume depletion or venous congestion? These findings can support vasopressor selection, fluid strategy, blood preparation, destination selection, and pre-arrival activation. They should not be used to exclude shock etiologies when images are poor.

BLUE-type lung ultrasound can support assessment of acute respiratory failure by looking for profiles consistent with pneumothorax, diffuse interstitial syndrome/pulmonary edema, focal consolidation, pleural effusion, or obstructive patterns (20). In transport, the practical version is not a full hospital protocol but a limited, bilateral, accessible-window assessment that answers whether a clear lung finding explains deterioration. Diffuse B-lines may support pulmonary edema or fluid intolerance, focal consolidation may support pneumonia or contusion, and pleural effusion may support drainage planning. These patterns are not disease-specific, so interpretation should remain integrated with history, vital signs, ventilation parameters, and receiving-hospital imaging.

Cardiac arrest POCUS can help identify selected reversible causes, such as tamponade, massive right ventricular dilation in suspected pulmonary embolism, profound hypovolemia, or pneumothorax when combined with lung views. It can also identify cardiac activity or standstill. However, cardiac arrest ultrasound is a high-risk use case because it may prolong chest-compression pauses. Adult advanced life support guidance emphasizes that POCUS must not cause additional or prolonged interruptions in compressions (28). For transport teams, cardiac arrest POCUS should be performed only by trained operators, only during planned rhythm or pulse checks, and only when the probe and screen are ready before the pause begins. The result should support reversible-cause management, not serve as the sole basis for termination decisions.

## 7 Ultrasound-guided procedures during transport

Transport ultrasound is not limited to diagnosis; it can also support selected procedures. Procedural feasibility depends strongly on staffing, space, sterility, vehicle motion, patient stability, and the urgency of the intervention (29–31). A procedure should be attempted during transport only when the expected benefit clearly outweighs the risk of distraction, delay, or procedural harm.

Ultrasound-guided vascular access is the most feasible procedural application. It may help with difficult peripheral venous access, long peripheral catheter placement, arterial access, or selection of a safer target before departure. In unstable patients with immediate need for access, ultrasound should not delay intraosseous access or other established emergency access routes. During interfacility retrieval, ultrasound-guided vascular access is often more feasible before loading or during a stable transport phase than in active motion. These applications are generally more feasible during pre-loading assessment, ED–ICU retrieval, or ground critical care transport than during active flight or highly unstable transport.

Thoracic procedures represent a second category. Lung ultrasound can help localize pneumothorax or pleural fluid and can support the decision to decompress or drain when the clinical context is compatible. However, it should not replace clinical decompression when tension pneumothorax is strongly suspected and the patient is unstable. For hemothorax or large effusion, ultrasound can inform timing and destination preparation, but definitive tube thoracostomy is often safer at the receiving facility unless transport physiology requires immediate intervention.

Pericardiocentesis and resuscitative thoracotomy are rare, high-risk procedures and should not be presented as routine transport ultrasound applications. Ultrasound may help identify tamponade physiology and activate a pathway, but invasive intervention during transport requires appropriate expertise, equipment, sterility, monitoring, and system authorization. Regional anesthesia and nerve blocks may be feasible in some retrieval systems, but they are not core emergency transport POCUS procedures and require local credentialing and governance.

## 8 Integrating POCUS into transport workflows

The main transport risk is not only a poor image; it is a wrong decision based on an uncertain image. A simple workflow can reduce this risk by defining indication, scope, timing, interpretation, escalation, and handover before the mission begins.

**Figure 1:**
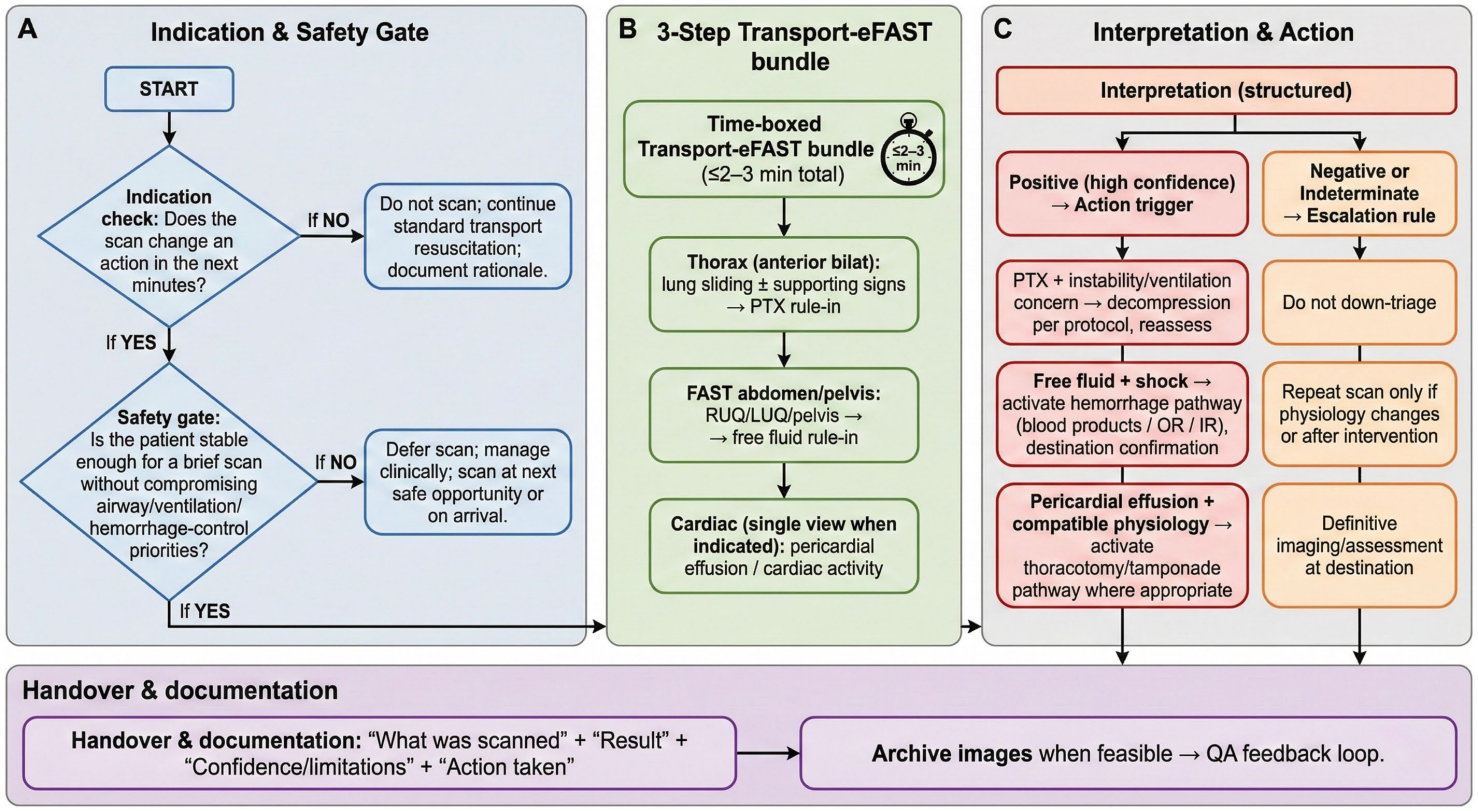
Safety-gated transport-eFAST workflow as one trauma-focused component of transport POCUS. **(A)** Indication and safety gate. **(B)** Three-step Transport-eFAST bundle. **(C)** Structured interpretation and action. The workflow begins with an indication check and a safety gate, proceeds to a time-boxed eFAST bundle only when scanning can change near-term action without compromising resuscitation, and ends with structured interpretation, action, handover, documentation, image archiving when feasible, and QA feedback.

Figure 1 summarizes a safety-gated Transport-eFAST workflow as one trauma-focused component of transport POCUS. The workflow begins with an indication check: will the scan change an action in the next few minutes? If not, the team should continue standard transport resuscitation and document the rationale for not scanning. If the scan could change action, a safety gate follows: is the patient stable enough for a brief scan without compromising airway, ventilation, circulation, or hemorrhage-control priorities? If not, scanning should be deferred until the next safe opportunity or until arrival.

When scanning is appropriate, the trauma bundle should be time boxed. A practical Transport-eFAST set includes anterior thoracic views for pneumothorax screening, RUQ/LUQ/pelvic FAST windows for free fluid, and a single cardiac view for pericardial effusion or cardiac activity when indicated (5, 6, 18, 19). Actionable positive findings should trigger predefined responses, whereas negative, unclear, or incomplete scans should activate escalation rules rather than reassurance. Handover should state what was scanned, the result, confidence and limitations, action taken, and whether images were archived for QA review.

## 9 Interfacility retrieval and CCATT-style long-range transport

After defining application-specific rules and workflow safeguards, the same principles can be applied across different transport models. For ED-ICU retrieval, ultrasound adds value when it answers a focused question that changes what the team does. Common questions include: Is pneumothorax or pleural fluid contributing to ventilation difficulty? Is pericardial effusion a concern? Which shock pattern is most likely when standard monitors are misleading? Is vascular access feasible before departure? Can findings improve pre-arrival coordination with the receiving ICU, trauma team, operating room, or interventional radiology suite (17, 19, 29–31)?

Interfacility transport guidelines emphasize structured processes, equipment readiness, staff competence, monitoring, and documentation as core safety elements (1–3). A systematic review of adult interfacility transport outcomes also highlights the need for better adverse event reporting (4). These principles should apply to transport POCUS. Ultrasound should be integrated into mission planning and handover, not added as an informal bedside test without governance.

CCATT-style teams deliver ICU-level care in austere air medical settings and may manage patients for prolonged periods when definitive imaging is unavailable (32, 33). In this setting, serial POCUS can support trend monitoring for lung congestion, pleural fluid, cardiac function, pericardial effusion, and response to interventions. The same safety rule applies: act on clear positives and avoid false reassurance from unclear negatives.

## 10 Governance, training, credentialing, and quality assurance

A transport ultrasound program is more than a device and a probe. It is a clinical system requiring a written scope, defined indications, training standards, credentialing, documentation, QA, and feedback (1–4, 17). Variation in HEMS POCUS availability, indications, training, and governance has been reported in Europe, which increases the risk of uneven scan quality and inconsistent downstream decisions (34).

Training should focus on competence and decision safety, not only case counts. Studies of eFAST performed by emergency physicians and newly trained clinicians show useful diagnostic accuracy, but false negatives still occur, especially when windows are limited or the team is rushed (18, 27, 35). This supports a rule-in approach. Operators should learn when not to scan, how to label indeterminate results, and how to communicate limitations.

Quality assurance should include discrepancy review against CT, operative findings, radiology reports, procedural outcomes, and receiving-team feedback. Programs should track scan time, indeterminate rate, image archiving rate, documentation completeness, false-negative events, false-positive actions, departure delay, and procedure-related complications. A continuous feedback approach in a UK HEMS program provides a model for safer scaling when direct supervision is not available during missions (36). Table 3 provides a governance checklist with patient-safety endpoints.

**Table 3:** Governance checklist for a transport ultrasound program.

Domain	Minimum standard	What to track	Common failure	Practical fix	Patient-safety endpoint
Scope of practice	Written indications and limits. Clear “confirm vs. exclude” rules.	Off-scope scan rate	Scope creep	Question-first scanning and stop rules (17).	Off-scope scan causing delayed transport, inappropriate procedure, or down-triage.
Protocol and stop rules	Time caps. “Indeterminate = unsafe to act on as reassurance.”	Scan time, indeterminate rate, departure delay	Delayed departure and distraction	Role assignment and strict time caps.	Delayed airway, hemorrhage-control, transport, or CPR tasks caused by scanning.
Training and credentialing	Competency-based sign-off and revalidation.	Credentialed staff rate, supervised scan completion, assessment results	Variable skill and false reassurance	Simulation, structured assessment, and remediation (17).	False-negative or false-positive action linked to operator skill gap.
Documentation	Structured report with result, confidence, limitation, and action.	Documentation completeness, image archive rate	Missing limitation and overconfidence	Templates with required fields (1, 17).	Receiving team acts on incomplete or overconfident handover.
Quality assurance and feedback	Regular review with discrepancy tracking.	Discrepancy rate vs. CT, operative findings, radiology, or clinical outcome; major discrepancy rate; time-to-feedback.	No learning loop	Continuous feedback model (36).	Missed injury, unnecessary procedure, or delayed escalation not captured for learning.
Tele-mentoring policy	Defined indications, roles, data handling, and dropout plan.	Tele-mentoring use, image quality, dropout rate, outcome of advice	Unclear responsibility	Protocol, fallback plan, QA, and escalation rules (37).	Remote advice unavailable or delayed without local fallback.
Procedure governance	Procedure list authorized by setting, operator, equipment, and environment.	Procedure success, complication rate, sterility breaches, delays	High-risk procedure attempted outside scope	Local credentialing and procedure-specific stop rules.	Procedure-related harm or delayed definitive care.

## 11 Technology-enabled transport POCUS

Tele-mentoring and streaming may expand the reach of transport ultrasound, but they also add failure points. A feasibility study of telementored eFAST in an ambulance helicopter found high remote expert image-quality ratings (mean 4.9/5) and a mean examination duration of 05:54 min, but LTE coverage depended on flight path and altitude, and audio problems required fallback communication (37). Tele-mentoring can help, but it cannot be the only plan. Teams need a fallback workflow, such as store-and-forward clips, still images, or phone-based discussion, while keeping the same strict rule-in thresholds.

Live video and dispatch-support systems may increasingly become part of transport decision-making. The SEE-IT feasibility randomized trial tested bystander video livestreaming for trauma incidents and focused on whether livestreaming could be activated, viewed, and used to support resource decisions without clear evidence of psychological harm (38). This work is not ultrasound-specific, but it shows how transport POCUS may eventually sit within a broader command-and-control workflow that includes live video, remote clinicians, image sharing, consent processes, data security, documentation, and audit trails (17, 38).

## 12 Patient-centered outcomes, safety endpoints, and research gaps

Patient-centered outcomes remain the major evidence gap. Many studies report feasibility, diagnostic accuracy, management change, or time-to-care effects, but few demonstrate reductions in mortality, complications, missed injury, ICU length of stay, ventilator days, transfusion needs, or long-term functional outcomes. This limitation is important because transport outcomes depend on many factors beyond ultrasound, including dispatch, staffing, destination resources, blood availability, and prehospital time intervals (4, 10, 12, 13, 16).

Safety endpoints need more attention. Future studies should track harm related to false reassurance, including delayed imaging, delayed surgical escalation, delayed decompression, inappropriate destination selection, or under-triage after a negative or indeterminate scan (17). Studies should also report unnecessary procedures after false-positive findings, prolonged CPR interruptions, departure delays caused by scanning, loss of sterility during procedures, and major discrepancies between transport POCUS and CT, surgery, radiology, or autopsy.

Future studies should standardize reporting of scan indication, operator training level, vehicle type, timing of the scan, scan duration, window success rate, indeterminate rate, image archiving, action triggered by positive scans, escalation after negative or unclear scans, and QA feedback. Pragmatic pathway trials and implementation studies should evaluate whether POCUS improves clinically meaningful outcomes beyond time-to-care, and whether tele-mentoring or AI-assisted image interpretation improves safety without increasing workload or delays (30, 31, 37, 38).

## 13 Conclusion

In trauma and critical care transport, POCUS should function as a structured decision-support tool. It can speed recognition of time-critical problems and support early coordination with receiving teams, but it should not replace definitive imaging. Its safest role is confirmation of actionable problems rather than exclusion of injury or disease. A rule-in approach fits the transport environment: actionable positive findings can trigger predefined responses, whereas negative, unclear, or technically limited scans should be treated as uncertain and should never justify down-triage. Safe scaling depends on clear scope, competency-based training, time caps, documentation of confidence and limitations, discrepancy review, and routine quality assurance.
